# The Antioxidant Activity of Polysaccharides Derived from Marine Organisms: An Overview

**DOI:** 10.3390/md17120674

**Published:** 2019-11-29

**Authors:** Qiwu Zhong, Bin Wei, Sijia Wang, Songze Ke, Jianwei Chen, Huawei Zhang, Hong Wang

**Affiliations:** 1 College of Pharmaceutical Science & Collaborative Innovation Center of Yangtze River Delta Region Green Pharmaceuticals, Zhejiang University of Technology, Hangzhou 310014, China; 1362546154@qq.com (Q.Z.); binwei@zjut.edu.cn (B.W.); new8090@hotmail.com (S.W.); 459282333@qq.com (S.K.); cjw983617@zjut.edu.cn (J.C.); hwzhang@zjut.edu.cn (H.Z.); 2 Center for Human Nutrition, David Geffen School of Medicine, University of California, Rehabilitation Building 32-21, 1000 Veteran Avenue, Los Angeles, CA 90024, USA

**Keywords:** antioxidant activity, marine organisms, polysaccharides, chemical composition, structural characteristics, structure–activity relationship

## Abstract

Marine-derived antioxidant polysaccharides have aroused extensive attention because of their potential nutritional and therapeutic benefits. However, the comprehensive comparison of identified marine-derived antioxidant polysaccharides is still inaccessible, which would facilitate the discovery of more efficient antioxidants from marine organisms. Thus, this review summarizes the sources, chemical composition, structural characteristics, and antioxidant capacity of marine antioxidant polysaccharides, as well as their protective in vivo effects mediated by antioxidative stress reported in the last few years (2013–2019), and especially highlights the dominant role of marine algae as antioxidant polysaccharide source. In addition, the relationships between the chemical composition and structural characteristics of marine antioxidant polysaccharides with their antioxidant capacity were also discussed. The antioxidant activity was found to be determined by multiple factors, including molecular weight, monosaccharide composition, sulfate position and its degree.

## 1. Introduction

Oxygen is a key substance in the normal metabolic activities of aerobic organisms [1]. In a high redox potential environment, the organism will inevitably produce reactive oxygen species (ROS), including hydroxyl radical (^•^OH), superoxide anion (O_2_^•^^-^), hydrogen peroxide (H_2_O_2_), nitroxide radicals (NO^•^), and peroxyl radicals (ROO^-^) [2]. ROS play an important role in various physiological and biochemical activities of organisms. The low or moderate concentration of ROS prevent the infectious agents from infecting the host cells and disturbs the cell mitosis [3], while the high concentration may destroy the balance of the prooxidants/antioxidants in the organism to cause oxidative stress [4]. The excessive ROS not only impair the nutritional value of food through oxidation before consumption, but also damage the normal function of cell lipids, proteins, and DNA to induce diseases [5], such as cancer, diabetes, inflammatory diseases, neurodegenerative diseases, aging, and immune system damage [6,7]. 

Oxidative stress is considered as an imbalance between the prooxidants and antioxidants in the body, and usually eliminated by antioxidant defense system [8], including many antioxidant enzymes and non-enzymatic antioxidants. Unfortunately, when there are more free radicals present than can be quenched by the body’s antioxidant defense system, hence, damage to tissues, and even diseases began to appear. The most effective and widely used strategy to reduce oxidative stress is to supplement exogenous antioxidants [9]. In recent years, there have been concerns over the safety of synthetic antioxidants, therefore antioxidants derived naturally are attracting more attention. Natural products, such as carotenoids, tocopherols, and flavonoids show strong antioxidant activity in scavenging free radicals and relieving cellular damage caused by oxidation and have been added in health supplements, food additives, and pharmaceuticals [10,11,12]. Another group of naturally-derived chemicals, polysaccharides, have also attracted wide attention because of their promising in vitro and in vivo biological activity [13,14,15,16,17]. 

Marine organisms have been considered as a promising source of nutrients and bioactive compounds [18,19]. In recent years, many polysaccharides from marine organisms with antioxidant activity have been isolated and identified, but the characteristics of these polysaccharides were rarely summarized and their structure-activity relationships were scarcely reported. This paper reviews the research progress in antioxidant polysaccharides derived from marine organisms, including their source, type, chemical components, structural characteristics and antioxidant capacity, and the protective in vivo effects mediated by antioxidative stress. Finally, the relationships between the chemical structure and antioxidant activity of these polysaccharides will also be discussed.

## 2. Marine-Derived Antioxidant Polysaccharides

**Figure 1:**
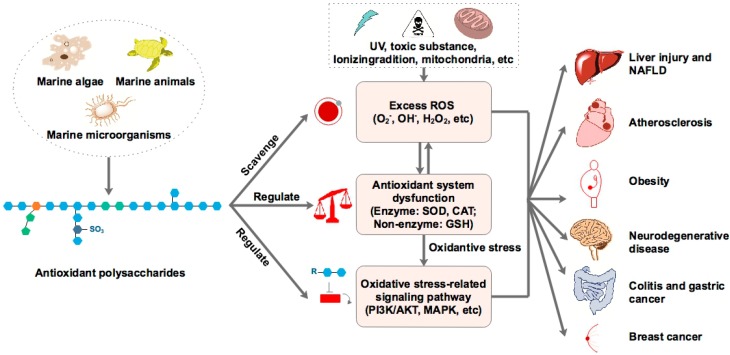
Overview of marine-derived polysaccharides in alleviating oxidative stress-mediated diseases.

In the past few years, many studies have revealed that polysaccharides derived from marine organisms exhibit antioxidant activity, which can scavenge 2,2-diphenyl-1-picrylhydrazyl (DPPH), hydroxyl radical, peroxyl radical, alkyl radical, H_2_O_2_, superoxide radical and ABTS radical, and exhibit reducing power ability. The polysaccharides are mainly derived from marine algae, followed by marine microorganisms and marine animals (Table 1). Data from individual studies also suggested that marine-derived antioxidant polysaccharides could alleviate the oxidative stress-mediated diseases, such as liver injury, diabetes, obesity, neurodegenerative disease, colitis, and breast cancer. The effect could be explained by three distinct mechanisms, including scavenging the ROS, regulating the antioxidant system or oxidative stress-mediated signaling pathways (Figure 1), implying the complicated interactions of marine-derived antioxidant polysaccharides in reducing the oxidative stress. 

### 2.1. Algal Polysaccharides

Marine algae are valuable sources of bioactive natural products including antioxidants, antitumor agents, and antibacterial agents [20,21]. In recent years, with the continuous development and utilization of marine resources, many polysaccharides with antioxidant activity have been discovered from algae such as brown, green, and red [22]. The chemical structure, monosaccharide composition, molecular weight, sulfate content, as well as the antioxidant capacity of these algal polysaccharides are summarized in Table 1. These algal polysaccharides usually have hepatoprotective, neuroprotective, and anti-diabetic activities, which are directly or indirectly related to their antioxidant properties. The potential mechanisms are presented in Table 2.

#### 2.1.1. Brown Algal Polysaccharides

Brown algal polysaccharides are mainly found in the form of fucan, and few in the form of alginate and laminarin. Fucans are usually defined as fucoidan for polysaccharides extracted from marine organisms [106]. The dominant polysaccharide, fucoidan, mainly composed of l-fucose and sulfate groups, has shown in vitro and in vivo antioxidant activity, including a significant improvement in free radical-mediated diseases.

DPPH free radical scavenging assay is widely used to evaluate the in vitro antioxidant activity of samples [107]. Polysaccharides provide hydrogen or electrons to DPPH free radicals to form stable molecules (DPPH-H) [108]. The electron-withdrawing group of polysaccharides and the specific structures activate the hydrogen atoms on sugar residues [109]. Many studies have found that sulfated polysaccharides derived from brown algae have strong DPPH free radical scavenging activity and reduction ability. For example, fucoidan F3 from *Undaria pinnatifida* with an average molecular weight of 27 kDa and the sulfate content of 25.19%, has moderate DPPH scavenging activity (68.65% at 1 mg/mL) (Table 1) [40,106]. Notably, polysaccharides from *Sargassum* usually exhibit a high DPPH free radical scavenging ability. For instance, the fucoidan isolated from *Sargassum cinereum* had a DPPH scavenging activity of 51.99% at the concentration of 80 µg/mL (ascorbic acid 85.9% as a positive control) (Table 1) [32]. In addition, the sulfated polysaccharide STP-1 from *Sargassum thunbergii*, with a molecular weight of 190.4 kDa and sulfate content of 15.2%, has a strong DPPH scavenging capacity (95.23% at 0.4 mg/mL) (Table 1) [36]. Furthermore, many brown algal polysaccharides also have strong ferrous ion-chelating and reducing power activities. The fucoidan from *Spatoglossum asperum* has a reducing power of 60.15% at a concentration of 0.1 mg/mL (Table 1) [37]. Moreover, the IC_50_ value of the fucoidan from *Sargassum glaucescens* to ferrus ion-chelating ability was 0.65 mg/mL (Table 1) [33]. These valuable findings indicate that brown algal polysaccharides have strong antioxidant activity, especially the ability to scavenge DPPH free radical. The high ratio of sulfate/fucose of these polysaccharides maybe responsible for the antioxidant activity [33,110].

**Figure 2:**
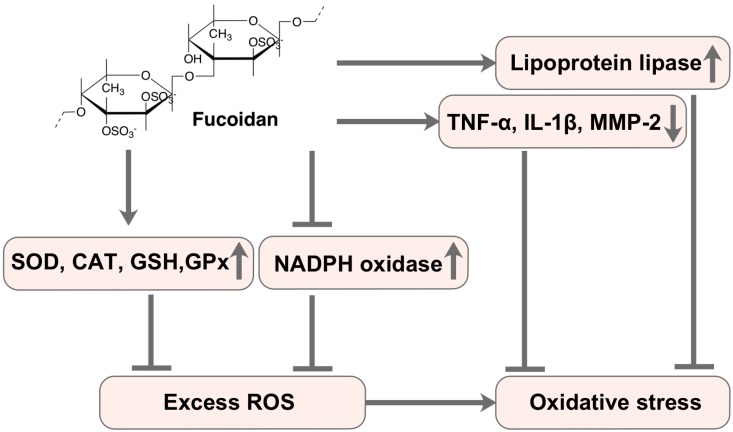
Fucoidan prevents oxidative stress by regulating the antioxidant system.

Numerous in vivo antioxidant studies have revealed that fucoidan derived from brown algae can alleviate body damage caused by oxidative stress through regulating the antioxidant defense system in the body (Table 2). For example, fucoidan from *Costaria costata* inhibits the oxidative stress in the liver of CCl_4_-induced mice by downregulating the malondialdehyde (MDA) level and upregulating the superoxide dismutase (SOD) level (Figure 2) [24]. Moreover, Meenakshi et al. [80] have documented that fucoidan extracted from *Turbinaria decurrens* reduces the levels of lipid peroxidation markers MDA and TBARS in alcoholic rats, and increases the levels of non-enzymatic antioxidant glutathione (GSH) and the enzymatic antioxidant SOD, CAT, and GPx in the liver, ultimately reduces the hepatic oxidative damage caused by alcohol **(**Figure 2). In addition, fucoidan not only reduces the liver MDA and NO concentration and upregulates the GSH level, but also reduces the mRNA expression of TNF-α, IL-1β, and MMP-2 to inhibit the production of ROS in the liver, thus alleviating the development of non-alcoholic fatty liver disease (NAFLD) and inhibiting insulin resistance induced by high fat diet [82]. 

Interestingly, fucoidan from *Undaria pinnatifida* can inhibit the apoptosis of PC12 cells and improve the cognitive ability in Alzheimer’s disease model mice by upregulating the expression of the apoptosis-inhibiting proteins by activating the SOD activity and increasing the GSH levels [85]. A recent study also found that fucoidan from *Laminaria japonica* can improve the atherosclerotic cardiovascular disease by reducing the production of ROS by inhibiting NADPH oxidase subunit 4 (Figure 2) [74]. Another study showed that fucoidan derived from *Cladosiphon okamuranus* Tokida significantly inhibits LDL-C peroxidation and hepatic steatosis in the apolipoprotein E-deficient mice by activating plasma lipoprotein lipase activity, thus alleviating the dyslipidemia and atherosclerosis in the mice (Figure 2) [71]. These studies indicate that fucoidan inhibits the generation of ROS in vivo and then relieves the oxidative damage in a variety of pathways.

**Figure 3:**
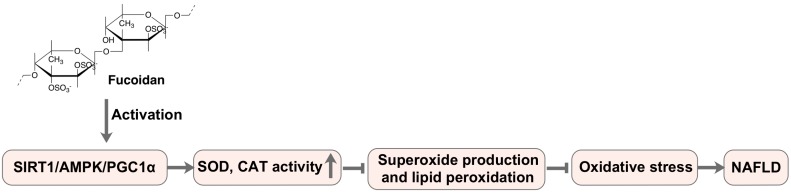
Fucoidan prevents oxidative stress through oxidative stress-related signaling pathway.

In addition to regulating the antioxidant defense system, fucoidan can also inhibit the ROS production through the oxidative stress-related signaling pathways, thereby alleviating the oxidative stress-related diseases. A recent study revealed that low molecular weight fucoidan derived from *Laminaria japonica* upregulates the activity of SOD and CAT by activating the SIRT1/AMPK/PGC1α signaling pathway, inhibiting the superoxide production and the lipid peroxidation, reducing TNF-α and transcription factor NF-κB, and then inhibiting oxidative stress in the liver and ultimately relieves NAFLD in diabetic mice (Figure 3) [78]. Furthermore, fucoidan derived from *Fucus vesiculosus* significantly increased the expression of MnSOD and decreased the ROS level through the AKT pathway, and finally enhanced the survival and angiogenesis of mesenchymal stem cells in the hind limb ischemia model [73]. Interestingly, Ryu and Chung [79] reported that fucoidan could upregulate the expression of HO-1 and SOD-1 genes by activating Nrf2, and then attenuate the oxidative stress in HaCaT cells. In conclusion, fucoidan derived from brown algae not only has significant in vitro antioxidant activity but also modulates the oxidative stress-mediated diseases by regulating the antioxidant defense systems and the oxidative stress-related signaling pathways in cellular and experimental animal models.

#### 2.1.2. Red Algal Polysaccharides 

In the past few decades, marine red algae have been extensively studied as a major source of agar. Recently, many red algal polysaccharides displayed good in vitro and in vivo antioxidant activity (Table 1). For instance, the IC_50_ value of polysaccharide AMG-LMWP extracted from *Pyropia yezoensis* for the H_2_O_2_ scavenging ability and alkyl free radical scavenging ability were 13.0, and 114.4 μg/mL, respectively (Table 1) [43]. However, compared with brown and green algae, red algal polysaccharides generally have lower DPPH and hydroxyl radical scavenging activity in vitro, which may be related to their lower uronic acid content [41].

Of note, polysaccharides derived from red algae alleviate the damage of the digestive tract organs and liver via the mediation of the oxidative stress (Table 2). Brito et al. [87] found that sulfated polysaccharide from *Gracilaria birdiae* reduced the colonic cell damage by inhibiting the decline of intestinal antioxidant GSH activity, preventing inflammatory cell infiltration and increasing MDA and NO_3_/NO_2_ levels. Likewise, a study has shown that polysaccharide SFP extracted from *Solieria filiformis* with in vitro ferrous ion chelation and hydroxyl radical scavenging activity has prevented ethanol-induced gastric cell damage by increasing the antioxidant GSH level [45]. Hence, red algal polysaccharides may prevent the disorder of the antioxidation system by upregulating the antioxidant GSH activity. Interestingly, Damasceno et al. [89] found that sulfated polysaccharide derived from *Hypnea musciformis* can activate K-ATP channels through NO, inhibit oxidative stress, and alleviate the ethanol-induced gastric injury in mice. 

ROS play an important role in hyperlipidemia and cellular senescence. The increase in ROS is often accompanied by an increase in the levels of TC and LDL-C, and ROS can also convert the excess LDL-C into oxidized LDL-C and promote the development of metabolic syndrome [111]. A study has shown that porphyran extracted from *Porphyra haitanensis* significantly increased the levels of antioxidant enzymes SOD and GSH-Px in the liver of hyperlipidemia mice, thereby inhibiting the increase of plasma HDL-C mediated by oxidative stress [112]. In addition, studies disclosed that red algal polysaccharides can alleviate cell damage and aging [113,114], but the possible protective mechanisms of red algal polysaccharides on cell damage are still unclear. Subsequently, studies revealed that oxidative stress and DNA damage could be relieved by red algal polysaccharides via reducing the activity of SA-β-gal and inhibiting p53–p21 pathways, and ultimately attenuating senescence of WI-38 cells induced by H_2_O_2_ [93].

An in vitro study revealed that sulfated polysaccharide ASPE extracted from *Laurencia papillosa* inhibits the proliferation of MDA-MB-231 human breast cancer cells by inhibiting the ratio of ROS and Bax/Bcl-2 protein levels in cells (Table 2) [90]. Moreover, carrageenans from the same source inhibited the proliferation and cell viability of MCF-7 human breast cancer cells by the same route in a dose-dependent manner (Table 2) [92]. In summary, red algal polysaccharides show protection against digestive organ damage, anti-aging, and anti-breast cancer effects mainly through the scavenging of excess ROS and the regulation of the antioxidant system.

#### 2.1.3. Green Algal Polysaccharides 

Green algae are an important traditional Chinese medicine used in the treatment of hydropic in China [115]. Green algal polysaccharides, usually known as ulvan, are mainly composed of α-l-rhamnose, xylose, glucuronic acid, iduronic, and sulfate [116]. Ulvan not only has strong in vitro antioxidant activity, but also abrogates the free radical-mediated diseases. Hence, green algae-derived antioxidant polysaccharides have recently aroused people’s attention.

Studies have shown that some ulvan exhibit strong superoxide and DPPH radical scavenging activity (Table 1). For example, ulvan from *Enteromorpha linza* have the EC_50_ value of 10.4 μg/mL for superoxide radical scavenging and an EC_50_ value of 0.84 mg/mL for DPPH radical scavenging (Table 1) [46]. In addition, ulvan from *Ulva fasciata* has a strong (81.45% at a concentration of 90 μg/mL) scavenging activity on superoxide radical (Table 1) [41]. The strong antioxidant activity of green algal polysaccharides may be attributed to the dominance of arabinogalactan [117].

A high lipid level in rats promotes the oxidative stress, leading to liver oxidative damage in the liver of rats [118]. However, ulvan derived from green algae can alleviate oxidative stress-mediated liver damage by increasing endogenous antioxidant enzyme activity, reducing oxidative stress (Table 2) [97,98,99]. A comparative study has shown that the higher sulfate content of polysaccharide HU extracted from *Ulva pertusa* can reduce the lipid peroxidation production by increasing the antioxidant CAT, SOD, and GSH-Px activity in the liver of hyperlipidemia rats, thus avoiding the corresponding liver tissue damage [99]. In addition, ulvan can also inhibit cell damage and liver cancer cell proliferation by regulating the antioxidant defense system of liver cancer rats induced by diethylnitrosamine. The regulation includes the improvement of the activities of antioxidant enzymes SOD, CAT, GR, MPO, GST, and the level of endogenous non-enzymatic antioxidant GSH [98]. In general, these high sulfate-rich ulvans have the potential to be developed as a drug for the prevention and treatment of liver damage.

The application of green algal polysaccharides in the food industry has also attracted people’s attention. Interestingly, the use of *Ulva fasciata*-derived polysaccharide to prepare edible films not only has better hydroxyl free radical scavenging activity but also better mechanical strength [119]. Therefore, green algae-derived antioxidant polysaccharides are expected to become green meat preservatives and food packaging materials. In summary, the green seaweed polysaccharides not only have potential as an antioxidant and antioxidant material for the food industry, but also has potential as a hepatoprotective agent for the pharmaceutical industry.

### 2.2. Microbial Polysaccharides 

The unique environment of the marine makes it become an abundant natural source of microbial extracellular polysaccharides (EPS) [120]. EPS produced by marine microorganisms have attracted attention due to their novel structure and biological activity. In recent years, many EPS from marine microorganisms have been found to have good antioxidant activity (Table 1), but their in vivo antioxidant activity were rarely reported.

#### 2.2.1. Microalgal Polysaccharides 

Microalgae such as *Chrysophyta*, *Bacillariophyta*, and *Pyrrophyta*, are easy to grow on a large scale and are good sources of aquatic animal feed and functional food materials [121,122]. Metabolites from microalgae are widely used as high-value products [123], such as antioxidative metabolites, carotenoids, xanthophylls, and phenolics [124,125]. 

Microalgal polysaccharides also showed potent activity toward different free radical scavenging [126,127,128]. Sun et al. [54] obtained two polysaccharides (P-0 and S-0) from algae *Pavlova viridis* and *Sarcinochrysis marina* Geitler, and found that the degraded polysaccharides (P-2 and S-3) showed stronger antioxidant activity than the undegraded polysaccharides (P-0 and S-0) (Table 1). The IC_50_ values of P-2 and S-3 to DPPH radical and hydroxyl free radical scavenging ranged from 0.41 to 0.45 mg/mL, which were stronger than polysaccharides derived from *Ecklonia cava* (IC_50_ value of 0.73 mg/mL) (Table 1) [26]. Luo et al. [129] found that *Spirulina platensis* polysaccharide has a strong antioxidant activity and could be used as an antioxidant in Sausage to extend its shelf life. Moreover, in view of the industrial scale culture of microalgae, microalgae EPS have the potential to become antioxidants in foods and cosmetics.

The high diversity of microalgal EPS are attributed to the regulation of polysaccharide biosynthesis in microalgae to adapt to different environmental conditions, which benefits the discovery of antioxidant polysaccharides and the investigation of their structural and activity relationships [124]. Fimbres-Olivarria et al. [53] obtained three sulfated polysaccharides (WSPN, RSPN, and BSPN) by cultivating *Navicula* sp. at three different wavelengths. The three polysaccharides have different molecular sizes, monosaccharide components, and total sugar content. Among them, WSPN has the highest DPPH free radical scavenging ability (IC_50_ value was 238 µg/mL) (Table 1). This indicates that different antioxidant microalgae EPS can be obtained by changing the different culture conditions of the microalgae. In addition, the IC_50_ value of the extracellular polysaccharide AEPS derived from *Graesiella* sp. on ferrous ion-chelating ability was 0.33 mg/mL (stronger than EDTA), and on hydroxyl radical scavenging was 0.87 mg/mL (ascorbic acid was 1.1 mg/mL) (Table 1). The strong antioxidant activity may be related to its structure, high uronic acid (24%) and high protein (12%) (Table 1) [51]. Furthermore, the in vivo antioxidant effects of microalgal EPS need to be further studied. These studies also suggest that microalgae are promising biological resources to produce antioxidant EPS.

#### 2.2.2. Fungal Polysaccharides

Marine fungi are one of the important sources of novel antioxidant polysaccharides. Wang et al. [56] found that marine fungus *Aspergillus terreus* produced a new extracellular polysaccharide (YSS) mainly consisting of mannose and galactose with a molecular weight of 18.6 kDa. YSS showed good scavenging capacity with the EC_50_ value of 2.8 mg/mL toward DPPH free radical scavenging capacity (Table 1). Chen et al. [58] found that the marine fungus *Fusarium oxysporum* produced a novel galactofuranose-containing exopolysaccharide Fw-1, which is mainly composed of galactose, glucose, and mannose with a molecular weight of 61.2 kDa. The EC_50_ values of Fw-1 on the scavenging of hydroxyl and superoxide radicals were 1.1 and 2.0 mg/mL, respectively, which is larger than that of the exopolysaccharide AVP isolated from marine *Aspergillus versicolor* LCJ-5-4 (EC_50_ value is 4.0 mg/mL) [130]. The characteristics and antioxidant activities of other EPS derived from a deep-sea *Aspergillus versicolor* N(2)bC [57] and sponge endophytic fungus *Alternaria* sp. [55] were summarized in Table 1. In general, antioxidant EPS derived from marine fungi tend to have a simpler monosaccharide composition and a smaller molecular weight, which makes it more suitable for the study of relationship between the structure and antioxidant activity of marine polysaccharides.

#### 2.2.3. Bacterial Polysaccharides

Exopolysaccharides produced by marine bacteria usually contribute to the formation of biofilm, thus adapting to the extreme environment of high salinity, low temperature, and high osmotic pressure, etc. [65]. In recent years, exopolysaccharides with strong antioxidant activity have been frequently found in marine bacteria [65,131,132].

Marine halophilic bacterial EPS have been concerned due to their novel characteristics and good antioxidant activity. Priyanka et al. [61] isolated a sulfated EPS mainly consisting of glucose and having a molecular weight of 269 kDa from the marine halophilic strain *Labrenzia* sp. PRIM-30. Its DPPH radical scavenging IC_50_ value is 0.64 mg/mL, and the hydroxyl radical scavenging IC_50_ value is 0.19 mg/mL (Table 1). Another study [64] found that the exopolysaccharide HMEPS secreted by the marine halophilic bacterium *Halolactibacillus miurensis*, mainly composed of galactose and glucose, has stronger DPPH free radical scavenging activity (IC_50_ value less than 0.10 mg/mL) than EPS from *Labrenzia* sp. PRIM-30. HMEPS also has a strong reducing ability that the superoxide radical scavenging capacity was 89.15% at the concentration of 0.5 mg/mL (Table 1). 

Increasing interest has been focused on the application of antioxidant EPS from marine bacteria in the fields of cosmetics, food, and medicine. Sun et al. [66] isolated a novel exopolysaccharide with good antioxidant activity from marine bacteria *Polararibacter* sp. SM1127 which exhibits a protective effect on human skin cells in a low-temperature environment and is safe for oral and external application. Recently, a similar study [60] revealed that an exopolysaccharide EPS-A isolated from *Aerococcus uriaeequi*, mainly composed of glucose and mannose, has a strong antioxidant activity. Its hydroxyl radical (45.65% at 0.1 mg/mL) and superoxide radical (67.31% at 0.25 mg/mL) scavenging activities were comparable to vitamin C (Table 1). It is noticeable that some EPS not only have antioxidant activity, but also inhibit the growth of cancer cells (Table 1 and Table 2) [62,63,132]. For instance, El-Newary et al. [62] found an exopolysaccharide (BAEPS) produced by the marine *Bacillus amyloliquefaciens* 3MS 2017, which contains 22.8% sulfate and has a molecular weight of 37.6 kDa. The IC_50_ values of BAEPS on radical scavenging of DPPH and hydrogen peroxide were 0.21 and 30.04 µg/mL, respectively. BAEPS also inhibits the growth of breast cancer cell MCF7 (IC_50_ value was 70 µg/mL). Moreover, marine actinomycetes-mediated EPS nanomaterials are arousing more and more attention in the pharmaceutical industry. Studies have found that EPS secreted by marine *Streptomyces violaceus* MM72 has antioxidant activity [59]. In addition, marine bacterial EPS can inhibit the formation of *Pseudomonas aeruginosa* and *Candida albicans* biofilm to inhibit the growth of drug-resistant bacteria [133,134]. These series of studies have shown that EPS derived from marine bacteria not only have the potential to develop into antioxidants, but also have the characteristics of the non-toxic, anticancer and anti-harmful microorganism biofilm. 

### 2.3. Animal Polysaccharides 

Antioxidant polysaccharides are not only rich in marine algae, but also have been found in marine animals, especially in marine invertebrates. Polysaccharides isolated from marine invertebrates show in vitro and in vivo antioxidant activities, thus could alleviate the diseases mediated by free radicals (Table 1 and Table 2).

Sea cucumber is a traditional Chinese medicine, widely circulated among the people. For the preparation of sea cucumber polysaccharides with antioxidant activity, a suitable extraction method is often required. Low-molecular-weight polysaccharide DHmG-3 with its primary structure and sulfate retained could be obtained by degrading *Holothuria mexicana* glycosaminoglycans (HmG) with H_2_O_2_/ascorbic acid, and it showed moderate scavenging ability to superoxide radicals and hydroxyl radicals, which was lower than DHmG (Table 1) [69]. The weak antioxidant activity of DHmG-3 may be explained by the remove of phenolic substance in HmG with the treatment of H_2_O_2_ [135,136]. In addition, the polysaccharides isolated from sea cucumber by the alkali extraction method have moderate antioxidant activity (Table 1) [67]. However, the polysaccharide PPP and its fractions from sea cucumber *Phyllophorus proteus* have comparable scavenging abilities of superoxide radical with vitamin C [137]. Similarly, the polysaccharide (HfP) extracted from *H. fuscogliva* by enzymatic hydrolysis method has similar hydroxyl radical scavenging activity with vitamin C, but the superoxide radical scavenging activity is stronger than vitamin C [68]. Of note, a novel polysaccharide (Ta-FUC) composed of tetrafluoro-recycling units was obtained from *Thelenota ananas* by enzymatic hydrolysis and possessed strong superoxide radical scavenging activity (the IC_50_ value of Ta-FUC and vitamin C was 17.46 and 132.64 µg/mL) (Table 1) [70]. Therefore, the sea cucumber polysaccharides prepared by the enzymatic hydrolysis method have strong antioxidant activity, in particular, have strong superoxide radical scavenging activity. This may be because of the presence of the sulfate group (more than 20%) increases the electron density of the carbon atoms and promotes the release of hydrogen, and thus increases the scavenging capacity of superoxide radicals [138]. Superoxide radicals are precursors of hydroxyl radicals and singlet oxygen, which can induce lipid peroxidation and oxidative damage of protein and DNA [137,139]. Therefore, sea cucumber polysaccharides prepared by enzymatic hydrolysis may have a potential protective effect on the diseases induced by superoxide radicals. 

*Sipunculus nudus* polysaccharide (SNP) prepared by alkali extraction not only has strong hydroxyl and superoxide scavenging ability, but also inhibits the hematopoietic bone marrow damage induced by radiation via reducing the nitric oxide levels and increasing the activity of antioxidant enzymes [103,140]. Similarly, SNPs with an average molecular weight of 680 kDa prepared by alkali extraction not only have strong scavenging activity against hydroxyl radicals (99% at 10 mg/mL), but also prevent radiation-induced bone marrow oxidative damage by increasing DNA content in bone marrow cells (Table 1 and Table 2) [104]. Therefore, alkali-extracted SNPs may play an important role in preventing radiation damage. 

## 3. Factors Affecting the Antioxidant Activity of Polysaccharides

### 3.1. Molecular Weight

Low molecular weight polysaccharides obtained by degrading high molecular weight polysaccharides exhibit higher antioxidant activity [141,142,143]. It has been reported that a 55.0 kDa polysaccharide P2 obtained by degrading the 3645 kDa polysaccharide P0 exhibits higher DPPH scavenging activity although they have comparable sugar and sulfate content and the same monosaccharide composition (Table 1) [54]. A recent study demonstrated that low molecular weight polysaccharides present a greater number of reducing ends that are used to react with free radical species, thus possess high antioxidant activity [48]. However, the polysaccharides with small molecular weight from the excessive degradation of marine polysaccharides have lower antioxidant activity than the original polysaccharides. Polysaccharides with too small molecular weight fail to form a suitable conformation to maintain the antioxidant activity [144]. It has been reported that a 9.83 kDa polysaccharide DHmG-3 obtained by degrading the 99.7 kDa polysaccharide HmG exhibits lower DPPH scavenging activity than HmG even though they have comparable sugar and sulfate content and the same monosaccharide composition (Table 1) [69]. Three different molecular weight polysaccharides (24, 15.1, 10.3 kDa) were obtained by the degradation of fucoidan (molecular weight 38.2 kDa) derived from *Fucus vesiculosus* for different times. With the decrease of molecular weight, the DPPH free radical scavenging activity of the polysaccharide tend to increase first and then decrease [135]. Interestingly, polysaccharides extracted from *Ulva prolifera* had different molecular weights (84.5–227 kDa) but similar protein (1.95–2.13%), sulfate (5.77–7.66%), and total sugar (78.3–84.07%) content, showing comparable ABTS scavenging activity (3.7–6.7%) and no DPPH scavenging activity [145]. These findings indicate that polysaccharides with suitable molecular weight may show better antioxidant activity, but the molecular weight range may differ from samples and more single factor comparative analysis is needed to understand the effect of molecular weight on the antioxidant activity of polysaccharides.

### 3.2. Monosaccharaides Composition 

Marine algal polysaccharides show high chemical composition diversity because of the difference in the harvest season, location, and species of marine algae, which is closely correlated with their biological activity [25,146]. The composition of the monosaccharide affects the antioxidant activity of the marine polysaccharides (Table 1). For example, polysaccharide P2 from *Pavlova viridis* [54] and polysaccharide UFP2 from *Ulva fasciata* [47] have comparable molecular weights (55.0 and 54.7 kDa, respectively) and sulphate content (17.80% and 16.28%, respectively), but have different monosaccharide composition, polysaccharide P2 mainly consists of glucose, rhamnose, D-fructose, and mannose, while polysaccharide UFP2 mainly consists of rhamnose, glucosamine, and xylose. Interestingly, P2 shows much stronger DPPH and hydroxyl radicals scavenging activities than that of UFP2 (DPPH, 96% vs. 20–25%; hydroxyl radicals: 98% vs. 40–45%) (Table 1). Fimbres-Olivarria et al. [53] isolated two polysaccharides (BSPN and RSPN) with comparable molecular weight (107 and 108 kDa, respectively) and sulfate content (0.33% and 0.32%, respectively) from *Navicula* sp. and similar monosaccharide composition but different monosaccharide contents (BSPN has higher galactose content than RSPN), resulting in a much higher DPPH free radical scavenging capacity of BSPN than RSPN (IC_50_ values of 326 and 3066 µg/mL, respectively) (Table 1). In addition, among the three polysaccharides from *Laminaria japonica*, polysaccharide LJPA-P3 with higher content of galactose (91.9%) has stronger ABT radical scavenging activity (70%), while the other two polysaccharides LJPA-P1 and LJPA-P2 with lower galactose content (28.7% and 54.5%, respectively) [147]. This finding indicates that the different amounts of the same type of monosaccharide may also affect the antioxidant activity of marine polysaccharides. In particular, polysaccharides with higher galactose content tend to have better antioxidant activity. However, how the monosaccharide composition affects the antioxidant activity of marine polysaccharides is still unclear.

### 3.3. Sulfation Degree and Position

The content and the position of sulfate of the marine polysaccharides affect their antioxidant activity. Many studies have shown that polysaccharides with high sulfate content tend to have stronger antioxidant activity [47,148,149]. Shao et al. [47] conducted that partial desulfation of marine-derived sulfated polysaccharides and found that the same polysaccharide with partial desulfurization has a significantly lower antioxidant activity than the undesulfurized polysaccharide, implying that the sulfate content affects the antioxidant activity of the polysaccharide. It may be because the presence of a sulfate group in the polysaccharide activates the hydrogen on the anomeric carbon, which enhances the hydrogen supply capacity of the polysaccharide, and thus its antioxidant activity is enhanced [150]. Secondly, the higher sulfate content can improve the water solubility and physicochemical characteristics of the polysaccharide, thus increasing the biological activity [151]. Of note, Xiao et al. [152] studied the effect of sulfated modification of polysaccharides SPP from *Sargassum pallidum* on their antioxidant activities, and found that the DPPH scavenging capacity of the modified polysaccharide S-SPP1-4 (sulphate 10.96%) was higher than unmodified SPP (sulphate 3.31%), however, an even higher sulfated modification of polysaccharide S-SPP1-8 (sulphate 13.46%) has lower DPPH scavenging capacity than that of S-SPP1-4. Liu et al. [153] also reported a consistent phenomenon. This may be because the further increase of the degree of sulfation breaks the triple-helical structure of the polysaccharide, thus affecting the ability of the polysaccharide to supply hydrogen for the antioxidant activity [153,154]. These findings indicate that polysaccharides with a higher degree of sulfation have greater antioxidant activity with the precondition that the triple-helical structures of polysaccharides are not destroyed.

**Figure 4:**
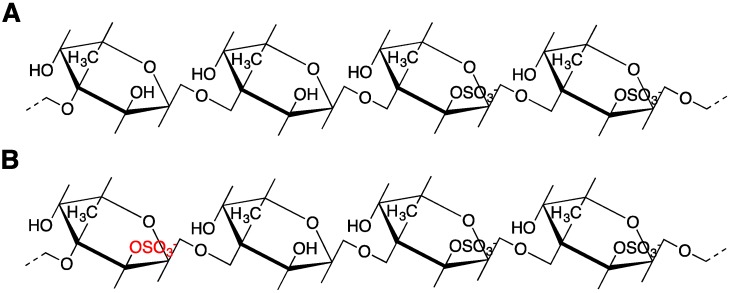
Structure units of fucoidan from sea cucumber (**A**) *Thelenota ananas* and (**B**) *Isostichopus badionotus*.

Sellimi et al. [155] found that sulfation at C-4 position of the polysaccharide has strong antioxidant activity (100% inhibition of DPPH at 1.5 mg/mL). Similarly, Mou et al. [67] extracted three sulfated polysaccharides from Chinese edible sea cucumber and found that C-4 sulfation polysaccharide showed the highest antioxidant activity. Furthermore, the sulfate derivatization of polysaccharides occurs mainly at the C-6 position, which also enhances the antioxidant activity of polysaccharides [149,156,157]. This may be since the sulfation of the C-4 and C-6 positions of the polysaccharide activates the hydrogen atom of the anomeric carbon and enhances the chelation, thereby promoting hydrogen supply capacity and preventing the generation of hydroxyl radicals [157]. However, Yu et al. [70] found that the only difference between the *Isostichopus badionotus* polysaccharide and the *Thelenota ananas* polysaccharide is that the former has an extra C-2 sulfation structure (Figure 4), and it displayed weaker antioxidant activity than the latter. This may be due to the sulfation of the C-2 position inhibits the supply of hydrogen to the polysaccharide. Thus, sulfate derivatization at the C-4 and C-6 positions of the polysaccharide can increase its antioxidant activity, and sulfate derivatization at the C-2 position may be reversed.

### 3.4. Others

Some small antioxidant molecules may remain in the polysaccharides, thus interfering the antioxidant activity determination. A study has shown that the antioxidant activity of algal polysaccharides is related to the presence of phenols and flavonoids [157,158], since many phenols and flavonoids show good antioxidant activity. This demonstrated that the purity of the antioxidant polysaccharides is crucial for their antioxidant capacity, and further studies are needed in this area. Notably, some studies have found that proteins or peptides may also act as polysaccharide conjugates to enhance the antioxidant activity of polysaccharides [159,160]. This may be due to the ability of some amino acids to donate protons to electron-deficient radicals. In addition, Burg et al. [161] found that the composition and concentration of salt affect the antioxidant activity of microalgal polysaccharides, of which Ca^2+^ has a strong reinforcing effect on the antioxidant activity of polysaccharides. This may be attributed to the interaction of salt ions with polysaccharides to make the conformational change, and then expose more antioxidant active sites [162].

Attention should also be paid to the slight difference of other factors of the polysaccharides when analyzing the contribution of a specific factor to their antioxidant capacity since it is a big challenge to prepare two polysaccharides with only one difference in chemical composition or structural characteristics. Therefore, the inconsistent of antioxidant polysaccharides in the structure–activity relationship maybe a result of the interaction of these factors. 

## 4. Conclusions and Perspective

In this paper, we summarized the sources, chemical composition, structural characteristics and antioxidant capacity of polysaccharides derived from marine organisms. We found that marine organism-derived antioxidant polysaccharides were mainly isolated from marine algae, especially from brown algae, followed by marine microorganisms and animals. Notably, marine algae-derived polysaccharides usually showed relatively higher antioxidant activity than those from marine microorganisms and animals. On the other hand, marine-derived polysaccharides not only have strong in vitro antioxidant activity, but also exhibit potent in vivo antioxidant capacity through scavenging ROS, regulating the antioxidant system or oxidative stress-mediated signaling pathways, thus alleviating oxidative stress-mediated diseases, such as liver injury, diabetes, obesity, neurodegenerative disease, colitis, breast cancer. These findings suggest that marine-derived antioxidant polysaccharides have the potential to develop as functional foods or adjuvant drugs for oxidative stress-mediated diseases. 

According to the extensive comparison studies, the structural characteristics of marine-derived polysaccharides, including molecular weight, monosaccharide composition, sulfation degree, and position, significantly affect their antioxidant activity. Additionally, the loose correlation of the activity with these factors implies that these factors may be interrelated and together determine the antioxidant activity of marine-derived polysaccharides. However, how these factors together determine the antioxidant activity of polysaccharides is not clear, which warrant further study. Notably, the antioxidant activity of contaminated small molecules, such as phenols and flavonoids, in polysaccharides needs to be excluded in further analysis. Moreover, more studies should be focused on preparation of high-purity polysaccharides and precise structure identification of polysaccharides as well as investigating the interrelated relationship between the chemical structure and antioxidant activity of marine polysaccharides. Since recent studies have revealed polysaccharides could alleviate oxidative stress by regulating gut microbiota composition [163,164,165,166,167] or activating gastrointestinal immune cells [168,169,170], and could also be degraded in the gastrointestinal tract, thus pass through the intestinal epithelial cells and enter the blood circulation [171,172,173,174]. Future mechanistic study should pay more attention to the interaction between gut microbiota and marine-derived antioxidant polysaccharides.

## Figures and Tables

**Table 1:** Antioxidant polysaccharides derived from marine organisms reported during the years 2013–2019.

Source	Chemical and Mono-Saccharide Composition (% or Molar Ratio)	Average Mw (kDa)	Sulfate Content (%)	Antioxidant Activity	Place of Origin	References
**Brown algae**						
*Chnoospora minima*	Proteins: 3.16 ± 0.50; Total phenolic: 4.83 ± 0.16; Fuc: Rha: Gal: Glu: Man: Xyl = 33.3: 3.7: 7.1: 29.6: 19.2: 7.2		11.80 ± 0.79	DPPH (IC_50_ = 3.22 µg/mL); Hydroxyl (IC_50_ = 48.35 µg/mL)	Galle, Sri Lanka	[23]
*Costaria costata*	**F1**: Uronic acid: 4.34; Fuc: Gal: Man: Xyl: Glc = 52.3: 4.2: 8.3: 4.5	19.8	11.4	Hydroxyl (63.3% at 10.0 mg/mL)	Dalian coast in Liaoning province of China	[24]
	**F2**: Uronic acid: 4.34; Fuc: Gal: Man: Xyl: Glc = 17.4: 7.6: 60.6: 6.8: 7.6	7.6	1	Hydroxyl (50.2% at 10.0 mg/mL)		
	**F3**: Uronic acid: 4.34; Fuc: Gal: Man: Xyl: Glc = 44.7: 15.9: 13.9: 21.1: 4.3	135.6	17.6	Hydroxyl (53.9% at 10.0 mg/mL)		
*Dictyota ciliolata*	Uronic acid: 13.94 ± 1.17; Total phenolic: 2.05 ± 0.18		5.44 ± 0.85	DPPH (27% at 2.0 mg/mL)	Puerto Morelos, Mexico	[25]
*Ecklonia cava*	Uronic acid: 11.3; Fuc: Ga: Xyl: Rha: Glu = 61.1: 27.2: 7.0: 3.9: 0.8	18 to 359 × 10^3^	20.1	DPPH (IC_50_ = 0.73 mg/mL); Peroxyl (IC_50_ = 0.48 mg/mL)	Jeju Island, South Korea	[26]
*Laminaria japonica*	**F1**: Uronic acid: 11.9; Fuc: Man: Glc: Rha: Arb: Glc-UA = 67.3: 14.3: 9.7: 0.3: 3.1: 5.2	28.95	17.77	DPPH (IC_50_ = 4.64 mg/mL)	China coast	[27]
	**F2**: Uronic acid: 2.01; Fuc: Man: Glc: Rha: Arb: Glc-UA = 54.7: 13.4: 5.8: 14.4: 11.6	46.17	30.38	DPPH (IC_50_ = 4.50 mg/mL)		
*Lobophora variegata*	Protein: 0.8; Gal: Xyl: Fuc = 36.8: 0.1: 29.2	35	32.6	DPPH (36.3% at 10 mg/mL)	Buzios	[28,29]
*Nemacystus decipients*	**HN0**: Uronic acid: 36.12 ± 0.97 Fuc: Xyl: Glu Fru: Man: Gal = 1: 0.15: 0.13: 0.13: 0.05: 0.05 (molar ratio)	1076 ± 30.27	20.37 ± 1.15	DPPH (IC_50_ = 3.96 mg/mL); Hydroxyl (IC_50_ = 4.12 mg/mL)	Jiangsu province, China	[30]
*Nemacystus decipients*	**HN1**: Uronic acid: 26.34 ± 1.27; Fuc: Xyl: Glu Fru: Man: Gal = 1: 0.27: 0.08: 0.10: 0.05: 0.05 (molar ratio)	886 ± 22.01	21.75 ± 1.10	DPPH (IC_50_ = 4.04 mg/mL); Hydroxyl (IC_50_ = 4.37 mg/mL)	Jiangsu province, China	[30]
	**HN2**: Uronic acid: 18.31 ± 0.76; Fuc: Xyl: Glu: Fru: Man: Gal = 1: 0.13: 0.02: 0.07: 0.04: 0.04 (molar ratio)	794 ± 19.52	22.03 ± 1.22	DPPH (IC_50_ = 3.88 mg/mL); Hydroxyl (IC_50_ = 3.07 mg/mL)		
	**HN3**: Uronic acid: 20.26 ± 1.06; Fuc: Xyl: Glu Fru: Man: Gal = 1: 0.19: 0.12: 0.02: 0.05: 0.02 (molar ratio)	676 ± 24.79	20.26 ± 1.06	DPPH (IC_50_ = 3.65 mg/mL); Hydroxyl (IC_50_ = 3.38 mg/mL)		
*Padinasanctae crucis*	Uronic acid: 11.87 ± 0.64; Total phenolic: 1.28 ± 0.05		5.18 ± 0.41	DPPH (22% at 2.0 mg/mL)	Puerto Morelos, Mexico	[25]
*Sargassum crassifolium*	**Extract1**: Uronic acid: 12.68 ± 0.25; Protein: 5.08 ± 0.32; Total phenolic: 3.52 ± 0.12	627.18 and 240.02	23.84 ± 0.08	DPPH (35-45% at 1.0 mg/mL); ABTS (75-80% at 0.2 mg/mL)	Pingtung, Taiwan province, China	[31]
	**Extract2**: Uronic acid: 15.83 ± 0.90; Protein: 3.05 ± 0.48; Total phenolic: 2.63 ± 0.16	628.97 and 237.26	23.59 ± 0.41	DPPH (35-45% at 1.0 mg/mL); ABTS (50-60% at 0.2 mg/mL)		
	**Extract3**: Uronic acid: 23.55 ± 1.99; Protein: 2.79 ± 0.17; Total phenolic: 2.77 ± 0.12	641.20 and 209.35	22.08 ± 0.55	DPPH (45-50% at 1.0 mg/mL); ABTS (50-60% at 0.2 mg/mL)		
*Sargassum cinereum*	Fuc: Gal: Man: Xyl = 65.7: 24.0: 3.5: 6.7		3.7 ± 1.54	DPPH (51.99% at 80 μg/mL)	Tuticorin coast, India	[32]
*Sargassum fluitans*	Uronic acid: 6.57 ± 0.30; Total phenolic: 1.83 ± 0.04		3.78 ± 0.65	DPPH (14% at 2.0 mg/mL)	Puerto Morelos, Mexico	[25]
*Sargassum glaucescens*	**SG1**: Uronic acid: 6.38; Protein: 3.78; Total phenolic: 2.70; Fuc: Xyl: Gal: Glu: Glu acid: Rha: Man = 1: 0.07: 1.24: 0.04: 0.28: 0: 0.68 (molar ratio)	690.8 and 327.1	6.38 ± 0.05	DPPH (IC_50_ = 4.30 mg/mL); Ferrous ion-chelating (IC_50_ = 0.65 mg/mL); Reducing (IC_50_ = 0.70 mg/mL)	Kenting, southern Taiwan	[33]
	**SG2**: Uronic acid: 27.99; Protein: 3.75; Total phenolic: 2.56; Fuc: Xyl: Gal: Glu: Glu acid: Rha: Man = 1: 0.05: 1.27: 0.05: 0.21: 0.0: 0.29 (molar ratio)	568.4 and 287.3	7.00 ± 0.06	DPPH (IC_50_ = 4.27 mg/mL); Ferrous ion-chelating (IC_50_ = 0.93 mg/mL); Reducing (IC_50_ = 0.60 mg/mL)		
	**SG3**: Uronic acid: 18.09; Protein: 2.76; Total phenolic: 2.02; Fuc: Xyl: Gal: Glu: Glu acid: Rha: Man = 1: 0.03: 1.09: 0.03: 0.10: 0: 0.29 (molar ratio)	636.9 and 280.4	6.67 ± 0.24	DPPH (IC_50_ = 4.57 mg/mL); Ferrous ion-chelating (IC_50_ = 4.09 mg/mL); Reducing (IC_50_ = 0.45 mg/mL)		
	**SG4**: Uronic acid: 11.42; Protein: 2.97; Total phenolic: 1.07; Fuc: Xyl: Gal: Glu: Glu acid: Rha: Man = 1: 0.05: 0.77: 0.02: 0.19: 0: 0.53 (molar ratio)	577.8 and 271.4	11.42 ± 0.03	DPPH (IC_50_ = 5.15 mg/mL); Ferrous Lon-chelating (IC_50_ = 1.04 mg/mL); Reducing (IC_50_ = 0.70 at 2.0 mg/mL)		
*Sargassum horneri*	**SHP30**: Glu: Rha: Man: Gal: Xyl = 24.95: 60.63: 8.09: 6.33: 0 (molar ratio)	1.58 × 10^3^	19.41	DPPH (85.01% at 2.5 mg/mL); Superoxide (65.0% at 2.5 mg/mL); Hydroxyl (98.07% at 2.5 mg/mL)	Zhejiang province, China	[34]
	**SHP60**: Glucose: Rha: Man: Gal: Xyl = 48.04: 32.19: 6.93: 10.01: 2.83 (molar ratio)	1.92 × 10^3^	13.15	DPPH (73.96% at 2.5 mg/mL); Superoxide (64.5% at 2.5 mg/mL); Hydroxyl (85.56% at 2.5 mg/mL)		
	**SHP80**: Glu: Rha: Man: Gal: Xyl = 100: 0: 0: 0: 0 (molar ratio)	11.2 × 10^3^	11.4	DPPH (71.74% at 2.5 mg/mL); Superoxide (35.0% at 2.5 mg/mL); Hydroxyl (47.57% at 2.5 mg/mL)		
*Sargassum polycystum*	Uronic acid: 3.9 ± 1.8; Fuc: Gal: Xyl: Glu: Rha: Man = 46.8: 14.3: 13.2: 11.5: 8.6: 5.6		22.35 ± 0.23	DPPH (61.22% at 1.0 mg/mL); Reducing (67.56% at 1.0 mg/mL); TAC (65.30% at 1.0 mg/mL)	The Gulf of Mannar region, Tamilnadu, India.	[35]
*Sargassum thunbergii*	**STP-1**: Protein: 1.86; Ara: Gal: Glu: Xyl: Man: GalA: GlcA = 1.9: 30.7: 4.5: 23.2: 17.6: 8.1: 13.9	190.4	15.2	DPPH (95.23% at 0.4 mg/mL); Hydroxyl (67.56% at 0.8 mg/mL)	Changdao, Shangdong province, China	[36]
	**STP-2**: Protein: 2.22; Ara: Gal: Glu: Xyl: Man: GalA: GlcA = 2.81: 23.2: 2.92: 20.8: 22.8: 9.74: 17.7	315.3	11.4	DPPH (90.80% at 0.4 mg/mL); Hydroxyl (68.7% at 0.8 mg/mL)		
*Spatoglossum asperum*	Protein: 4.2 ± 0.56; Fuc: Gal: Man: Rha: Xyl = 60.9: 25.2: 4.2: 6.3: 3.4		21.35 ± 0.81	DPPH (52.30% at 0.1 mg/mL); Reducing (60.15% at 0.1 mg/mL)	Tamil Nadu, India	[37]
*Turbinaria conoides*	**TCFE**: Uronic acid: 12.2		22.7	DPPH (IC_50_ = 534.45 µg/mL); ABTS (IC_50_ = 323.8 µg/mL)	Tuticorin coast, India	[38]
*Turbinaria ornata*	Uronic acid: 11.42 ± 0.03; Protein: 1.81 ± 0.035; Total phenol: 6.16 ± 0.36		27 ± 1.49	ABTS (IC_50_ = 88.71 µg/mL); DPPH (IC_50_ = 440.07 µg/mL); Superoxide (IC_50_ = 352 µg/mL)	Tamil Nadu, India	[39]
*Undaria pinnatifida*	**F1**: Uronic acid: 4.34; Fuc: Gal: Xyl: Glc: Man = 48.5: 37.9: 3.7: 2.9: 7.0	81	6.96	DPPH (53.45% at 1.0 mg/mL)	Great Barrier Island, New Zealand	[40]
	**F2**: Uronic acid: 0.84; Fuc: Gal: Xyl: Glc: Man = 53.2: 42.1: 1.2: 1.3: 2.2	22	22.78	DPPH (58.65% at 1.0 mg/mL)		
	**F3**: Uronic acid: 0.67; Fuc: Gal: Xyl: Glc: Man = 59.7: 28.7: 1.6: 2.8: 7.2	27	25.19	DPPH (68.65% at 1.0 mg/mL)		
	**F4**: Uronic acid: 4.34; Fuc: Gal: Man: Xyl: Glc = 46.6: 17.0: 11.6: 17.2: 7.6	80.3	23.5	Hydroxyl (59.1% at 10.0 mg/mL)		
**Red algae**						
*Gloiopeltis furcata*	Uronic acid: 1.35; Protein: 2.30; Rha: Xyl: Glu: Fru: Gal: Fuc = 0.35: 0.2: 0.66: 0: 0: 0.8: 0 (molar ratio)		24.1	Superoxide (64.37% at 90 µg/mL); DPPH (23.49% at 0.1 mg/mL)	NanjiArchipelago coast of China	[41]
*Gracilaria rubra*	**GRPS-1-1**: Uronic acid: 1.82 ± 0.06; Protein: 0.16 ± 0.04; Fuc: Gal = 1: 1.8 (molar ratio)	1310	5.96 ± 0.91	DPPH (41.59% at 2.5 mg/mL); Superoxide (64.78% at 2.5 mg/mL); ABTS (59.01% at 2.5 mg/mL)	Dayang Foodstuff Co.; Ltd	[42]
	**GRPS-2-1**: Uronic acid: 1.40 ± 0.09; Protein: 0; Fuc: Gal = 1: 2.16 (molar ratio)	691	8.46 ± 0.75	DPPH (30.67% at 2.5 mg/mL); Superoxide (50.47% at 2.5 mg/mL); ABTS (47.55% at 2.5 mg/mL)		
	**GRPS-3-1**: Uronic acid: 1.52 ± 0.13; Protein: 0; Fuc: Gal = 1: 2.76 (molar ratio)	923	12.03 ± 0.80	DPPH (22.84% at 2.5 mg/mL); Superoxide (64.28% at 2.5 mg/mL); ABTS (50.49% at 2.5 mg/mL)		
*Pyropia yezoensis*	**AMG-HMWP**: Gal: Glc: Man = 92.3: 4.0: 3.7	909.5 to 71.70	0.7	Alkyl (IC_50_ = 191.4 µg/mL); H_2_O_2_ (IC_50_ = 91.0 µg/mL)	Wando Island coast of South Korea	[43]
	**AMG–LMWP**: Gal: Glc: Man = 27.3: 64.5: 8.3	3.93 to 0.60	0.9	Alkyl (IC_50_ = 114.4 µg/mL); H_2_O_2_ (IC_50_ = 13.0 µg/mL)		
	**AMG hydrolysates**: Gal: Glc: Man = 93.6: 4.6: 1.8		0.9	Alkyl (IC_50_ = 197.5 µg/mL); H_2_O_2_ (IC_50_ = 95.0 µg/mL)		
*Sarcodia ceylonensis*	Man: Glc: Sor: Ara = 14.4: 5.3: 2.8: 1.2 (molar ratio)	466		Hydroxyl (83.33% at 4 mg/mL); ABTS (IC_50_ = 3.99 mg/mL)	Antarctic algae Co. (Xiamen, China)	[44]
*Solieria filiformis*	Total sugar: 66.0	210.9	6.5	DPPH (88.93% at 4.0 mg/mL); ABTS (IC_50_ = 2.01 mg/mL)	Atlantic coast, northeast of Brazil	[45]
**Green algae**					
*Enteromorpha linza*	**WP**: Uronic acid: 14.4; Rha: Xyl: Man: Glc: Gal = 3.4: 1: 0.35: 0.29: 0.15 (molar ratio)		21.3	DPPH (EC_50_ = 0.84 mg/mL); Superoxide (EC_50_ = 10.4 µg/mL)	Coast of Ningbo, China	[46]
	**AP**: Uronic acid: 20.5; Rha: Xyl: Man: Glc: Gal = 2.4: 1: 0.23: 0.21: 0.18 (molar ratio)		17.4	DPPH (EC_50_ = 0.96 mg/mL); Superoxide (EC_50_ = 15.6 µg/mL)		
*Ulva fasciata*	**UFP1**: Uronic acid: 0.19; Protein: 0.15; Rha: Xyl: Glc = 8.21: 1.53: 0.68	1.9	22.03	Superoxide (39.88% at 8 mg/mL); Hydroxyl (45-50% at 1 mg/mL)	Coast of Nanji Archipelago, China	[47]
	**UFP2**: Uronic acid: 4.46; Protein: 0.53; Rha: Xyl: Glc = 72.47: 4.59: 10.28	54.7	16.28	Superoxide (73.74% at 8 mg/mL); Hydroxyl (40-45% at 6 mg/mL);DPPH (20-25% at 1 mg/mL)		
	**UFP3**: Uronic acid: 18.36; Protein: 0.16; Rha: Xyl: Glc = 57.41: 24.25: 8.10	262.7	13.31	Superoxide (43.08% at 8 mg/mL); Hydroxyl (40-45% at 1 mg/mL)		
*Ulva fasciata*	Uronic acid: 35.06; Rha: Xyl: Glu: Fru: Gal: Fuc = 35.21: 17.81: 8.64: 0: 0: 0: 0 (molar ratio)		19.41	Superoxide (81.45% at 90 µg/mL); DPPH (37.63% at 0.1 mg/mL)	Nanji Archipelago coast of China	[41]
*Ulva intestinalis*	**Water extraction**: Protein: 0.48–0.63; Ara: Glu: Rha = 0: 11.86: 12.7	300	34–40	DPPH (56.18% at 3.0 mg/mL); ABTS (68.06% at 3.0 mg/mL)	Pattani Bay, Thailand	[48]
	**Acid extraction**: Protein: 0.9–2.96; Ara: Glu: Rha = 0.74: 0.84: 8.29	110	36–38	DPPH (>50% at 3.0 mg/mL); ABTS (71.87% at 3.0 mg/mL)		
	**Alkaline extraction**: Protein: 3.53– 3.97; Ara: Glu: Rha = 0: 11.96: 39.24	88	36–40	DPPH (55.97% at 3.0 mg/mL); ABTS (61.01% at 3.0 mg/mL)		
*Ulva intestinalis*	**FSP30**: Sulphate: Sugar: 1.08: 1 (molar ratio)	110	8.85	DPPH (the highest 82.23%)	Pattani Bay, Thailand	[49]
**Microalgae**						
*Odontella* *aurita* K-1251	**CL1**: No protein or nucleic acid; Glu: Man: Rib: Ara: Xyl: Gal = 82.2:13.3: 0.5: 3.6:0.3: 0.16	7.75		DPPH (42.45 % at 0.1 mg/mL); Hydroxyl (83.54 % at 10 mg/mL)	Copenhagen, Denmark	[50]
*Graesiella* sp.	Protein: 12; Uronic acid: 24; Fuc: Gal: Ara: Glc: Man: Xyl: Rib: Rha = 32: 16.3: 12.5: 12.1: 11.5: 10.3: 2.7: 2.3		11	Hydroxyl (IC_50_ = 0.87 mg/mL); Ferrous ion-chelating (IC_50_ = 0.33 mg/mL)	A hot spring located in the N-E of Tunisia	[51]
*Isochrysis galbana*	**IPSII**: Uronic acid: 25.6; Heteropolysaccharide	15.93	54.9	Superoxide (53.5% at 3.2 mg/mL)	Ocean University of China	[52]
*Navicula* sp.	**WSPN**: Protein: 1.65 ± 0.10; Glu, Rha: Gal: Man: Xyl = 15.46: 35.34: 24.48: 4.89: 9.28	17	0.40 ± 0.004	DPPH (IC_50_ = 238 µg/mL)	University of Sonora	[53]
	**BSPN**: Protein: 0.48 ± 0.001; Glu: Rha: Gal: Man: Xyl = 29.23: 10.67: 21.37: 4.43: 5.18	107	0.33 ± 0.004	DPPH (IC_50_ = 326 µg/mL)		
	**RSPN**: Protein: 0.55 ± 0.03; Glu: Rha: Gal: Man: Xyl = 17.41: 19.81: 16.82: 5.07: 10.38	108	0.32 ± 0.002	DPPH (IC_50_ = 3066 µg/mL)		
*Pavlova viridis*	**P0**: Uronic acid: 3.46 ± 0.24; Rha: Ara: Fru: Glu: Man = 6.63: 0.0: 21.9: 60.8: 10.6	3645	16.6 ± 0.37	DPPH (IC_50_ = 0.77 mg/mL); Hydroxyl (IC_50_ = 0.70 mg/mL)	Ocean University of China	[54]
	**P1**: Uronic acid: 5.88 ± 0.48; Rha: Ara: Fru: Glu: Man = 0: 0: 20.3: 75.9: 3.8.	387	15.0 ± 1.08	DPPH (IC_50_ = 0.56 mg/mL); Hydroxyl (IC_50_ = 0.52 mg/mL)		
	**P2**: Uronic acid: 8.78 ± 0.33; Rha: L-Ara: D-Fru: Glu: Man = 35.9: 0: 12.5: 50.1: 1.52.	55	17.8 ± 0.88	DPPH (IC_50_ = 0.45 mg/mL); Hydroxyl (IC_50_ = 0.42 mg/mL)		
*Sarcinochrysis marina* Geitler	**S0**: Uronic acid: 5.82 ± 0.53; Rha: L-Ara: D-Fru: Glu: Man = 0: 42.6: 8.81: 48.6: 0	2595	16.1 ± 0.75	DPPH (IC_50_ = 0.91 mg/mL); Hydroxyl (IC_50_ = 0.91 mg/mL)	Ocean University of China	[54]
	**S1**: Uronic acid: 9.21 ± 1.01; Rha: L-Ara: D-Fru: Glu: Man = 0: 33.2: 11.3: 55.3: 0	453	14.0 ± 1.08	DPPH (IC_50_ = 0.62 mg/mL); Hydroxyl (IC_50_ = 0.56 mg/mL)		
	**S2**: Uronic acid: 9.99 ± 0.49; Rha: L-Ara: D-Fru: Glu: Man = 0: 12.1: 32.9: 53.9: 0	169	17.3 ± 0.56	DPPH (IC_50_ = 0.51 mg/mL); Hydroxyl (IC_50_ = 0.48 mg/mL)		
	**S3**: Uronic acid: 0.03 ± 0.02; Rha: L-Ara: D-Fru: Glu: Man = 0: 21.4: 34.4: 44.2: 0	8.69	25.4 ± 0.69	DPPH (IC_50_ = 0.41 mg/mL); Hydroxyl (IC_50_ = 0.41 mg/mL)		
**Fungi**						
*Alternaria* sp. SP-32	**AS2-1**: Protein: 2.04; Not sulfate ester and uronic acid; Man: Glu: Gal = 1: 0.67: 0.35 (molar ratio)	27.4	0	DPPH (EC_50_ = 3.4 mg/mL); Hydroxyl (EC_50_ = 4.2 mg/mL)	South Sea, China	[55]
*Aspergillus terreus*	**YSS**: Protein and uronic acid not detected; Man: Gal = 88.5: 11.5	18.6	0	Hydroxyl (EC_50_ = 2.8 mg/mL)	Yellow Sea, China	[56]
*Aspergillus versicolor* N(2)bC	**N1**: Gal: Glu: Man = 2.46:1.49:1 (molar ratio)	20.5		Superoxide (EC_50_ = 2.20 mg/mL); DPPH (EC_50_ = 0.97 mg/mL)		[57]
*Fusarium oxysporum*	Protein: 0.79; Gal: Glu: Man = 1.33: 1.33: 1 (molar ratio)	61.2	0	Hydroxyl (EC_50_ = 1.1 mg/mL); Superoxide (EC_50_ = 2.0 mg/mL); DPPH (EC_50_ = 2.1 mg/mL)	South Sea, China	[58]
*Streptomyces violaceus* MM72	Man: Glu: Gal = 1.26:1.11:1.01 (molar ratio); Uronic acid: 10	8.96 × 10^5^		DPPH (IC_50_ = 76.38 mg/mL); Superoxide (IC_50_ = 67.85 mg/mL)	Tuticorin coast, India	[59]
**Bacteria**						
*Aerococcus uriaeequi*	**EPS-A**: Man: Glu = 1: 9.65			Hydroxyl (45.65% at 100 μg/mL); Superoxide (67.31% at 250 μg/mL)	Yellow Sea of China	[60]
*Alteromonas* sp. PRIM-21	**EPS**: Uronic acid: 46.60 ± 1.11; Protein: 6.34 ± 0.09; Acetyl: 1.86 ± 0.03; Phosphate: 0.22 ± 0.01		1.95 ± 0.04	DPPH (IC_50_ = 0.61 mg/mL); Superoxide (IC_50_ = 0.65 mg/mL)	Between Someshwara and Malpe, India	[61]
*Bacillus amyloliquefaciens* 3MS 2017	**BAEPS**: No protein or nucleic acid; Uronic acid: 12.3; Glu: Gal: GlcA = 1.6: 1: 0.9 (molar ratio)	37.6	22.8	DPPH (IC_50_ = 0.21 µg/mL); H_2_O_2_ (IC_50_ = 30.04 µg/mL); Superoxide (IC_50_ = 35.28 µg/mL)	Marsa-Alam	[62]
*Bacillus thuringiensis*	Fru: Gal: Xyl: Glu: Rha: Man = 43.8: 20.0: 17.8: 7.2: 7.1: 4.1			DPPH (79 % at 1.0 mg/mL); Superoxide (75.12 % at 1.0 mg/mL)	Campbell bay, India	[63]
*Enterobacter* sp. PRIM-26	**EPS**: Uronic acid: 25.33 ± 0.61, Protein: 6.34 ± 0.09; Acetyl: 1.17 ± 0.09; Phosphate: 0.11 ± 0.01		0	DPPH (IC_50_ = 0.44 mg/mL); Superoxide (IC_50_ = 0.33 mg/mL)	Between Someshwara and Malpe, India	[61]
*Halolactibacillus miurensis*	**HMEPS**: Gla: Glu = 61.87: 25.17			DPPH (84 % at 10 mg/mL); Superoxide (89.15 % at 0.5 mg/mL); Hydroxyl (61 % at 3.2 mg/mL)	Tuticorin, Southeast coast of India	[64]
*Haloterrigena turkmenica*	Glu: Glucosamine: GlcA: Gal: Galactosamine = 1: 0.65: 0.24: 0.22: 0.02; Uronic acid: 12.05	801.7 and 206.0	2.8	DPPH (IC_50_ = 6.03 mg/mL)	Braunschweig, Germany	[65]
*Labrenzia* sp. PRIM-30	Glu: Ara: GalA: Man = 14.4: 1.2: 1: 0.6 (molar ratio); Protein: 10.52 ± 0.9; Uronic acid: 2.26 ± 0.80	269	4.36 ± 0.68	DPPH (IC_50_ = 0.64 mg/mL); Superoxide (IC_50_ = 0.19 mg/mL)	Offshore of Cochin, India	[61]
*Nitratireductor* sp. PRIM-24	**EPS**: Uronic acid: 21.87 ± 0.50; Protein: 11.99 ± 0.15; Acetyl: 0.74 ± 0.02; Phosphate: 0.75 ± 0.02		2.20 ± 0.02	DPPH (IC_50_ = 0.49 mg/mL); Superoxide (Not scavenging activity)	Between Someshwara and Malpe, India	[61]
*Polaribacter* sp. SM1127	**EPS**: Little nucleic acid or protein; Rha: Fuc: GlcA: Man: Gal: Glc: N-Acetylglucosamine = 0.8: 7.4: 21.4: 23.4: 17.3: 1.6: 28.0	220		DPPH (55.40% at 10 mg/mL); Hydroxyl (52.1% at 10 mg/mL)	Ny-Ålesund, Svaldbard	[66]
**Animal**						
*Acaudina molpadioidea*	**fCS-Am**: GlcA: GalNAc: Fuc = 0.82: 1: 0.88 (molar ratio)	93.3	3.04	DPPH (65.9% at 4.0 mg/mL); Nitric oxide (39.3% at 4.0 mg/mL)	Fujian province, China	[67]
*Apostichopus japonicus*	**fCS-Aj**: GlcA: GalNAc: Fuc = 0.98: 1: 1.15 (molar ratio)	98.1	3.65	DPPH (48.1% at 4.0 mg/mL); Nitric oxide (25.9% at 4.0 mg/mL)	Nansha Islands of Nanhai Sea, China	[67]
*Holothuria fuscogliva*	**HfP**: Man: Rha: GlcA: Glc: Gal: Xyl: Fuc = 0.0836: 0.437: 0.134: 0: 1.182: 0.748 (molar ratio)	1.8671	20.7	Hydroxyl (EC_50_ = 3.74 mg/mL); Superoxide (EC_50_ = 0.0378 mg/mL)		[68]
*Holothuria mexicana*	**HmG**: GlcA: GalNAc: Fuc = 0.92: 1.00: 1.38 (molar ratio)	99.7	3.21	DPPH (65-70 % at 4 mg/mL); Superoxide (45-50 % at 4 mg/mL); Hydroxyl (60-65 % at 4 mg/mL)	Weifang city, China	[69]
	**DHmG-3**: GlcA: GalNAc: Fuc = 0.81: 1.00: 1.23 (molar ratio)	9.83	3.11	DPPH (50-55 % at 4 mg/mL); Superoxide (35-45 % at 4mg/mL); Hydroxyl (55-60 % at 4 mg/mL)		
*Stichopus chloronotus*	**fCS-Sc**: GlcA: GalNAc: Fuc = 0.90: 1: 1.08 (molar ratio)	111	3.18	DPPH (68.3% at 4.0 mg/mL); Nitric oxide (34.7% at 4.0 mg/mL)	Xisha Islands, China	[67]
*Thelenota ananas*	**Ta-FUC**: Novel tetrafucose repeating	1284	28.2 ± 3.5	Superoxide (IC_50_ = 17.46 µg/mL)	Hainan, China	[70]

AAPH: 2,2’-Azobis(2-amidinopropane) dihydrochloride; ABTS: 2,2’-Azino-bis(3-ethylbenzothiazoline-6-sulfonic acid); Ara: Arabinose; Fuc: Fucose; Gal: Galactose; GalA: Galacturonic acid; Glu: Glucose; GalNAc: N-acetylgalactosamine; GlcA: Glucuronic acid; Man: Mannose; Rha: Rhamnose; Xyl: Xylose.

**Table 2:** Protective effects and mechanisms of antioxidant polysaccharides derived from marine organisms.

Source	Polysaccharides	Test Model	Protective Effect	Potential Mechanism	References
**Brown algae**					
*Cladosiphon okamuranus* Tokida	Fucoidan	Apolipoprotein E-deficient mice	Anti-atherosclerosis	LPL activity↑, 4-HNE↓, MDA content↓, lipid peroxidation level↓	[71]
*Costaria costata*	Fucoidan	CCl_4_-induced liver injury in mice	Hepatoprotective	MDA content↓, SOD activity↑	[24]
*Dictyota ciliolata*	Fucoidan	HepG2 cells	Antioxidant in vivo	ROS level↓, GSH level↑, CAT activity↑	[25]
*Ecklonia cava*	Fucoidan	AAPH-induced oxidative stress in zebrafish model	Antioxidant in vivo	ROS level↓, Lipid peroxidation levels↓, cell death↓	[26]
	Fucoidan	Ultraviolet B-Irradiated mice	Anti-Photoaging	MDA content↓, ROS level↓, GSH level ↑	[72]
*Fucus vesiculosus*	Fucoidan	Mesenchymal stem cells and Murine hindlimb ischemia model	Anti-ischemic disease	ROS level↓, MnSOD level↑, GSH level↑, DNA damage↓, p38, JNK and caspase-3↓	[73]
*Laminaria japonica*	Fucoidan	Low density lipoprotein receptor-deficient (LDLR-/-) mice	Antiatherosclerosis	NOX4↓, ROS level↓	[74]
	Fucoidan	Diabetic goto-kakizaki rats	Anti-diabetic	eNOS expression and NO production↓,	[75]
*Laminaria japonica* Aresch	Fucoidan	The gentamicin induced nephrotoxicity in rats	kidney protection	AOPP and MDA levels↓, GSH level↑	[76]
*Laminaria japonica* Aresch	Fucoidan	STZ-induced type 1 diabetic rats	Anti-diabetic	ROS level↓, SOD activity↑, GSH level↑	[77]
*Laminaria japonica* Areschoug	Fucoidan	NAFLD in diabetes/obesity mice PA-treated HepG2 cells	Hepatoprotective	Hepatic CAT and SOD activity↑, MDA content↓ TNF-α and IL-6 level↓	[78]
*Lobophora variegata*	Galactofucan	Hepatotoxicity induced by CCl_4_ rats	Hepatoprotective	MPO activity↓, lipid peroxidation level↓	[29]
Marine brown algae	Fucoidan	HaCaT cells	Antioxidant in vivo	Nrf2 levels↑, HO-1, SOD-1 activity↑	[79]
	Fucoidan	Ethanol intoxicated Wistar rats	Hepatoprotective	GSH level↑, ROS level↓, TBARS level↓, SOD, CAT and GPx activity↑, Caspase3 expression↓	[80]
	Fucoidan	Cerebral ischemia reperfusion injury Sprague-Dawley rats	Neuroprotection	SOD and MDA levels↓, IL-1β, IL-6, MPO and TNF-α levels↓, p-p38 and p-JNK levels↓	[81]
	Fucoidan	HFD-induced NAFLD rats	Hepatoprotective	Hepatic MDA and NO levels↓, GSH↑, IL-1β and MMP-2 levels↓	[82]
*Padina sanctae-crucis*	Fucoidan	HepG2 cells	Antioxidant in vivo	ROS level↓, GSH level↑, CAT activity↑	[25]
*Sargassum crassifolium*	Fucoidan	H_2_O_2_-treated PC-12 cells	Neuroprotection	The sub-G_1_ DNA populations↓, the S phase populations↓	[31]
*Sargassum fluitans*	Fucoidan	HepG2 cells	Antioxidant in vivo	ROS level↓, GSH level↑, CAT activity↑	[25]
*Sargassum fusiforme*	Fucoidan	D-Gal-treated ICR mice	Anti-aging	SOD and CAT activities↑, MDA content↓, protein levels of Nrf2, Bcl-2, p21 and JNK1/2↑, Cu/Zn-SOD, Mn-SOD and GPX1 activity↑	[83]
*Turbinaria decurrens*	Fucoidan	MPTP-treated C57BL/6 mice	Neuroprotection	DOPAC, and HVA content↑, TBARS level↓, SOD and CAT activity↓, GSH level↑, GPX levels↑, TH and DAT protein levels↑	[84]
*Undaria pinnatifida*	Fucoidan	D-Gal-Induced neurotoxicity in PC12 cells and cognitive dysfunction in Mice	Neuroprotection	SOD activity↑, GSH level↑, ACh and ChAT activity↓, AChE activity↑	[85]
*Undaria pinnatifida* sporophylls	Fucoidan	Full-thickness dermal excision rat model	Promoting Wound Healing	MDA content↓, CAT and SOD activity↑, GSH level↑, lipid peroxidation level↓	[86]
**Red algae**					
*Gloiopeltis furcata*	Sulfated polysaccharides	H_2_O_2_-induced oxidative injury in PC12 cells	Anti-aging	ROS level↓, lipid peroxidation↓	[42,87]
*Gracilaria birdiae*	Sulfated polysaccharides	Trinitrobenzenesulfonic acid-induced colitis in rats	Anti-colitis	GSH level↑, MDA content↓, NO_3_/NO_2_ content↓, MPO activity↓, IL-1β and TNF-α levels↓	[87]
*Gracilaria cornea* Agardh	sulphated agaran	6-OHDA-treated Wistar rats	Neuroprotection	DA and DOPAC content↑, GSH ↑, iNOS and IL1β mRNA levels↓, NO_2_/NO_3_ levels in brain↑	[88]
*Hypnea musciformis*	Sulfated polysaccharides	Ethanol-induced gastric damage in mice	Gastroprotective	GSH levels↑, MDA content↓, NO levels↑	[89]
*Laurencia papillosa*	Sulfated polysaccharide ASPE	MDA-MB-231 human breast cancer cells	Anti-breast cancer	ROS level↓, Bax/Bcl-2 protein level ratio↓, cleaved caspase-3 protein level↓	[90]
	Sulfated carrageenan	MDA-MB-231 human breast cancer cells	Anti-breast cancer	Caspase-8 levels↑, caspase-3, caspase-9, p53 protein level ratio↓	[91]
	Carrageenans	MCF-7 human breast cancer cells	Anti-breast cancer	Bax/Bcl-2 protein level ratio↓, p53 and caspase-3 protein level↓	[92]
*Porphyra haitanensis*	Porphyran	H_2_O_2_-induced premature senescence in WI-38 cells	Anti-aging	SA-β-gal activity↓, p53 and p21 level↓	[93]
*Solieria filiformis*	Iota-carrageenan	Ethanol-induced gastric injury in mice	Gastroprotective	ROS level↓, GSH level↑, MDA content↓	[45]
*Chlorella pyrenoidosa*	Ulvan	MPTP-treated C57BL/6J mice	Neuroprotection	Contents of DA, DOPAC and HVA↑, ratio of DOPAC and HVA to DA↓, TNF-α, IL-1β and IL-6 levels↓	[94]
*Ulva fasciata*	Ulvan	Hyperlipidemia rats	Hepatoprotective	MDA content↓	[95,96]
*Ulva intestinalis*	Ulvan	J774A.1 cell	Immunostimulation	TNF-α levels↑, NO production↑, IL-1β expression↑	[49]
*Ulva lactuca*	Ulvan	D-galactosamine induced liver damage in rats	Hepatoprotective	Lipid peroxide level↓, DNA damage↓, SOD and CAT activities↑	[97]
	Ulvan	DiethylnitrosamineInitiated and phenobarbital-promoted hepatocarcino genesis in rats	Hepatoprotective	ROS level↓, MDA content↓, hepatic GSH, SOD, CAT, GR, MPO, and GST activity↑	[98]
*Ulva pertusa*	Ulvan	Cholesterol-rich diet rats	Hepatoprotective	MDA content↓, CAT, SOD and GSH-Px activity↑	[99]
	Ulvan	Hyperlipidemic Kunming mice	Hepatoprotective	MDA content↓, CAT and SOD activity↑,	[100]
**Microalge**					
*Spirulina platensis*	Polysaccharides	MPTP-treated C57BL/6J mice	Neuroprotective	SOD and GPx activity in serum and midbrain↑,	[101]
**Animal**					
*Sea cucumber*	Polysaccharides	Hyperlipidemia mice	Antihyperlipidemic	CAT and SOD activity↑, MDA content↓	[102]
*Sipunculus nudus*	Animal polysaccharides	Beagle dogs exposed to γ-radiation	Anti-radiation hematopoiesis	SOD activity↑	[103]
	Animal polysaccharides	A half-lethal dose 137Cs –rays irradiation mice	Anti-radiation hematopoiesis	SOD and GSH-P_X_ activity↑, MDA content↓	[104]
*Stichopus japonicus*	Animal polysaccharides	6-OHDA-exposed SH-SY5Y cells	Neuroprotective	MDA content↓, SOD activity↑, ROS level↓, NO release↓, Bax/Bcl-2 protein level ratio↓, levels of p-p53, p-p65, p-p38, JNK1/2, iNOS↓	[105]

AAPH: 2,20-Azobis(2-amidinopropane) dihydrochloride; ACh: Acetylcholine; AChE: Acetylcholine esterase; CAT: Catalase; CCl_4_: Carbon tetrachloride; ChAT: Choline acetyl transferase; Cu/Zn-SOD: Copper-zinc superoxide dismutase; DA: Dopamine; DAT: Dopamine transporter; D-Gal: D-galactose; DOPAC: 3,4-Dihydroxyphenylacetic acid; GR: Glutathione reductase; GSH: Glutathione; GSHPx: Glutathione peroxidase; 4-HNE: 4-hydroxynonenal; H_2_O_2_: Hydrogen peroxide; HVA: Homovanillic acid; IL-1β: Interleukins-1 beta; iNOS: Inducible nitric oxide synthase; JNK: Jun N-terminal kinase; MAO: Monoamine oxidase; MDA: Malondialdehyde; MMP-2: Mitochondrial membrane potential-2; MnSOD: Manganese superoxide dismutase; MPTP: 1-methyl-4-phenyl-1,2,3,6-tetrahydropyridine; MPO: Myeloperoxidase; NAFLD: Non-alcoholic fatty liver disease; NF-κB: Nuclear factor-κB; NO: Nitric oxide; NOX2: Cytochrome b-245β chain; Nrf2: Nuclear factor erythroid 2-related factor 2; 6-OHDA: 6-hydroxydopamine; PC12 cells: Rat pheochromocytoma cell line; ROS: Reactive oxygen Resource; SA-β-gal: Senescence-associated β-galactosidase; SH-SY5Y: Human neuroblastoma cell line; SOD: Superoxide dismutase; SOD-1: Superoxide dismutase-1; TBARS: Thiobarbituric acid reactive substances; TH: Tyrosine hydroxylase; TNF-α: Tumor necrosis factor α.
